# Supplementary material for: Patterns of Intron Gain and Loss in Fungi
Source: PLoS Biol. 2004 Nov 30;2(12):e422. doi: 10.1371/journal.pbio.0020422 (PMC532390; doi:10.1371/journal.pbio.0020422)
Supplement: Table S1 — Also available at http://genes.mit.edu/NielsenEtAl/. (4.3 MB ZIP). [file pbio.0020422.st001.zip › NielsenEtAl/html/622.html]

AN8485.1.NCU04315.1.MG00776.1.FG10144.1


```
 CLUSTAL W (1.82) Multiple Sequence Alignments - Introns Inserted


Sequence 1: AN8485.1	310 aa
Sequence 2: FG10144.1	331 aa
Sequence 3: NCU04315.1	333 aa
Sequence 4: MG00776.1	327 aa
Alignment Length: 350 aa
Number Identitical Residues: 115 aa
Alignment Score (without introns) 5805


MG00776.1 	MSPAT----VTIDTTPDFSNGGAPHS----TLLLAPPAMAAQQDAMAAALTDAAPLGSRP
NCU04315.1	MSPITL--DLTSDFNPANTTGAGSSSSQPRTLLVAPPSVASHEERISALFSTYP--RDTT
FG10144.1 	MAPATITIDNSDDFVMPVTKSAQTTSPPKRTLLLAPPSLAANSDALSTVLADYD--RSVT
AN8485.1  	MAPSFVSIDTTPDFDMTPTSSKSADSG-KRTLLLAPPSIATQEDKLRTLFTTYD--RSTT
          	*:*   : . : *     :..  . *    ***:***::*::.: : : ::      . .

MG00776.1 	ELYMLDRLAAGLTNLAPAAYDLVLVLASG-------ADPLLSRRDVFAKIADAMKPGAKL
NCU04315.1	DLHMLDRLAAGLVTLPTSTYDLILVLTDPDGSRHAEASALLSNRAVWSLLVPALKAGGKL
FG10144.1 	DLQMLDRLSVGLVTLPSSTYDLVLILSDAS-SKLDESLTLMN-RTVLGPVAESLKPSGRL
AN8485.1  	DLQMLDRVSAGFVSLPANTYDLVLVLTGTDGTRRSEALQLLK-REVYAAVVPAMKGGAKL
          	:* ****::.*:..*.. :***:*:*:. ..:   .:  *:. * * . :. ::* ..:*

MG00776.1 	RSRDGSP---LDAAIAKEAILAGLVESAEGAYEKPAE-EVAAVPLKFLKKKNKGEGGGPV
NCU04315.1	RSEDGTLGRDTTTPEAREAVLAGLVAGADGFTKPDYA-EEEAVPLRFGLKRKTNPN--PV
FG10144.1 	QSQNGNS--LEEASLSKEAVLAGLVSSHGGFEKPDYGDNDGAVTLKFG-KKKAQPA--PL
AN8485.1  	QTED-NF--FGEAE-DREAVLAGLVKKETGFEKMDVG-NGAAVPLRLGRKKKAAPA--PA
          	::.: .      :   :**:*****    *  :     :  **.*::  *::      * 

MG00776.1 	QSPAAATQPTAPAPAAAAKSLPNGVVMVDLNDDFDVS----DDEMIDEDELMTEEDLMRP
NCU04315.1	VAP---IQPVAQVVTAAPAGV--GFVTLDLNDDLDLDGEDDDDDVIDEDTLLTEADLRRP
FG10144.1 	ADGSVPLNHKRKPVEATPKPIVPAGVGF-IDLEDDLD----DDDLIDEDTLMTEEDLARP
AN8485.1  	P-------------VVQPPPIISS-------DDNDLN----DDELIDEDTLLSADDLKRP
          	               . .  :  .        : *:.    **::**** *::  ** **

MG00776.1 	IQQP1PECAPKPGKKRRACKDCTCGLAERLEAQDKARRDKADKQLQEKAATPFKLASEDL
NCU04315.1	IQQP~PECQPKPGKKRRACKDCTCGLAERLEAEDKARRDKADQALNT-----LKLKSEDL
FG10144.1 	IDIP1AECLPKAGKRRRACKDCTCGLAERLAAEDADKRATADKQLES-----IKLATDDL
AN8485.1  	IVPP1PECQPKAGKRRRACKDCTCGLAAQIEAEDRERREAADKSLNV-----MKLESDDL
          	*  * .** **.**:************ :: *:*  :*  **: *:      :** ::**

MG00776.1 	NELDFTVQGKTGSCGSCALGDAFRCAD~CPYIGLPAFKPGEEVTILNNVAQL
NCU04315.1	LELDLTVPGKTGSCGSCALGDAFRCAG1CPYLGLPPFKVGEEVSILNNVPQL
FG10144.1 	AEIDFTVQGKVGSCGNCSLGDAFRCDG~CPYIGLPPFKPGEEVRLLNNDVQL
AN8485.1  	NELDFTVQGKTGSCGNCALGDAFRCDG~CPFIGLPAFKPGQEVQILNDVAQL
          	 *:*:** **.****.*:******* . **::***.** *:** :**:  **
```
